# The longitudinal study of the relationship between social participation pattern and depression symptoms in frail older adults

**DOI:** 10.3389/fpsyt.2024.1440641

**Published:** 2024-09-03

**Authors:** Congqi Liu, Ruihao Zhou, Xilin Peng, Xudong Chen, Zhen Xia, Wei Wei, Tao Zhu, Guo Chen

**Affiliations:** ^1^ Department of Anesthesiology, National Clinical Research Center for Geriatrics, West China Hospital, Sichuan University, Chengdu, China; ^2^ The Research Units of West China (2018RU012)-Chinese Academy of Medical Sciences, West China Hospital, Sichuan University, Chengdu, China; ^3^ West China School of Medicine, Sichuan University, Chengdu, Sichuan, China

**Keywords:** frailty, social participation, depression symptom, latent class analysis, response surface analysis

## Abstract

**Background:**

Mental health challenges are encountered by frail older adults as the population ages. The extant literature is scant regarding the correlation between depressive symptoms and social participation among frail older adults.

**Methods:**

This study is based on an analysis of data from China Health and Retirement Longitudinal Study (CHARLS) participants aged 60 and older who are frail. A frailty index (FI) was developed for the purpose of assessing the frailty level of the participants. Additionally, latent class analysis (LCA) was employed to classify the participants’ social engagement patterns in 2015 and 2018. The study used ordered logistic regression to examine the relationship between social participation type and depressive symptoms. We also used Latent Transition Analysis (LTA) methods to explore the impact of changes in social activity types on depressive symptoms after three years of follow-up in 2018. In addition, the response surface analysis (RSM) investigation explored the relationship among FI, depression, and social participation.

**Results:**

A total of 4,384 participants completed the baseline survey; three years later, 3,483 were included in the follow-up cohort. The baseline survey indicates that female older adults in rural areas who are single, have lower incomes, shorter sleep durations, and lighter weights exhibited more severe depressive symptoms. Social participation patterns were categorized into five subgroups by LCA. The findings indicate that individuals classified as “board game enthusiasts” (OR, 0.62; 95% CI, 0.47-0.82) and those as “extensive social interaction” (OR,0.67; 95% CI, 0.49-0.90) have a significantly lower likelihood of developing depressive symptoms compared to the “socially isolated” group. We also discovered that “socially isolated” baseline participants who transitioned to the “helpful individual” group after three years had significantly greater depressed symptoms (OR, 1.56; 95% CI, 1.00-2.44). More social activity types and less FI are linked to lower depression in our study.

**Conclusion:**

The results of the study emphasize the importance of social participation patterns and the number of social participation types in relation to the severity of depression among frail older adults individuals. This study’s findings may provide important insights for addressing depressive symptoms in frail older adults person.

## 1 Introduction

The analysis of the Global Burden of Disease study indicates that there is presently an upward trend in global healthy life expectancy, indicating an acceleration of population aging worldwide (1). The duration that the older adults remain in a frailty status is proportionally extended as the average life expectancy rises. Frailty is a prevalent age-related syndrome characterized by a deterioration in the ability to respond to stimuli and the functioning of the body’s physiological systems (2). The most recent systematic review and meta-analysis study, which gathered extensive information from 240 studies in 62 countries, revealed an overall prevalence of frailty of approximately 18% (3). Older adults with frailty are more likely to have pathophysiological changes and mental and psychological abnormalities, which are strongly associated with cognitive decline, depression, disability and other diseases (4–6). Research suggests that frailty is typically chronic and dynamic in nature (7). Consequently, early intervention for frail older individuals has the potential to significantly improve their prognosis, reduce the occurrence of related complications, and improve their quality of life.

Depression is a common mental illness and one of the sources of mental distress for older adults in their later years. According to statistics, nearly 14% of people over the age of 55 worldwide suffer from depression, and 2% of them meet the diagnostic criteria for major depression (8). Late-life depression primarily manifests physical symptoms such as loss of energy, appetite changes or sleep disorders and mental symptoms such as sadness and negativity (9–12) Previous studies have shown that depression is positively correlated with the incidence of heart disease, stroke, diabetes and other geriatric diseases (13–17). Recent studies have suggested that the incidence of recurrent depression is significantly higher among old individuals, reporting an 18% higher rate compared to younger populations (18). Moreover, existing literature highlights the frequent co-occurrence of depressive symptoms and signs of frailty in older adults (5, 19, 20). Specifically, over one in ten individuals aged 55 and older adults are reported to exhibit concurrent symptoms of both conditions (21). These findings suggest that depression and frailty may act as interactive risk factors for each other.

Social interaction is widely recognized as an important protective factor for depression, and this conclusion has been confirmed in a large body of literature. Studies by Wang and Noguchi have consistently shown that increased social engagement correlates with significantly lower levels of depression among respondents (22, 23). Previous studies have particularly highlighted the critical role of social engagement in improving the physical and mental health of older adults (24–27). However, it should be noted that not all types of social activities have a positive impact on depressive symptoms. Several studies have pointed out that specific types of social activities may have a negative impact on depressive symptoms (28–30). Frail older adults individuals often face challenges such as mobility issues, chronic illnesses, or lack of familial support, which could limit their social engagement opportunities. Previous studies have indicated that frequent social participation can reduce the risk of frailty among Chinese older adults (31). Nevertheless, whether social engagement benefits older adults already experiencing frailty remains uncertain.

To our knowledge, there is an absence of research that has investigated the relationship between social participation type and the risk of incident depression in frail older adults. The primary purpose of this investigation was to ascertain whether the type of social participation has an impact on the level of depression among frail older adults. Additionally, we aim to investigate how changes in the types of social participation among frail older adults over time impact their depressive symptoms. For this purpose, data from a longitudinal cohort study conducted across the nation called the China Health and Retirement Longitudinal Study (CHARLS) were utilized.

## 2 Materials and methods

### 2.1 Study sample

The participants of our research were CHARLS-participating older adults aged 60 to 101. CHARLS is a nationally representative longitudinal cohort study. CHARLS collected high-quality data using face-to-face interviews and structured questionnaires, employing a four-stage stratified cluster sampling method. This approach recruited individuals aged 45 and older adults from 450 villages and community residents across 150 counties in 28 provinces throughout China. The initial nationwide survey (Wave 1) was carried out in 2011, with a total of 17,708 participants. Subsequent follow-up surveys, known as Wave 2, Wave 3, and Wave 4, were undertaken in 2013, 2015, and 2018 respectively. Detailed reports on the entire sampling process can be found elsewhere (32, 33). Peking University’s biomedical ethics review committee (IRB00001052-11015) approved the design and methodology of the CHARLS study, and informed consent was obtained from all participants.

**Figure 1:**
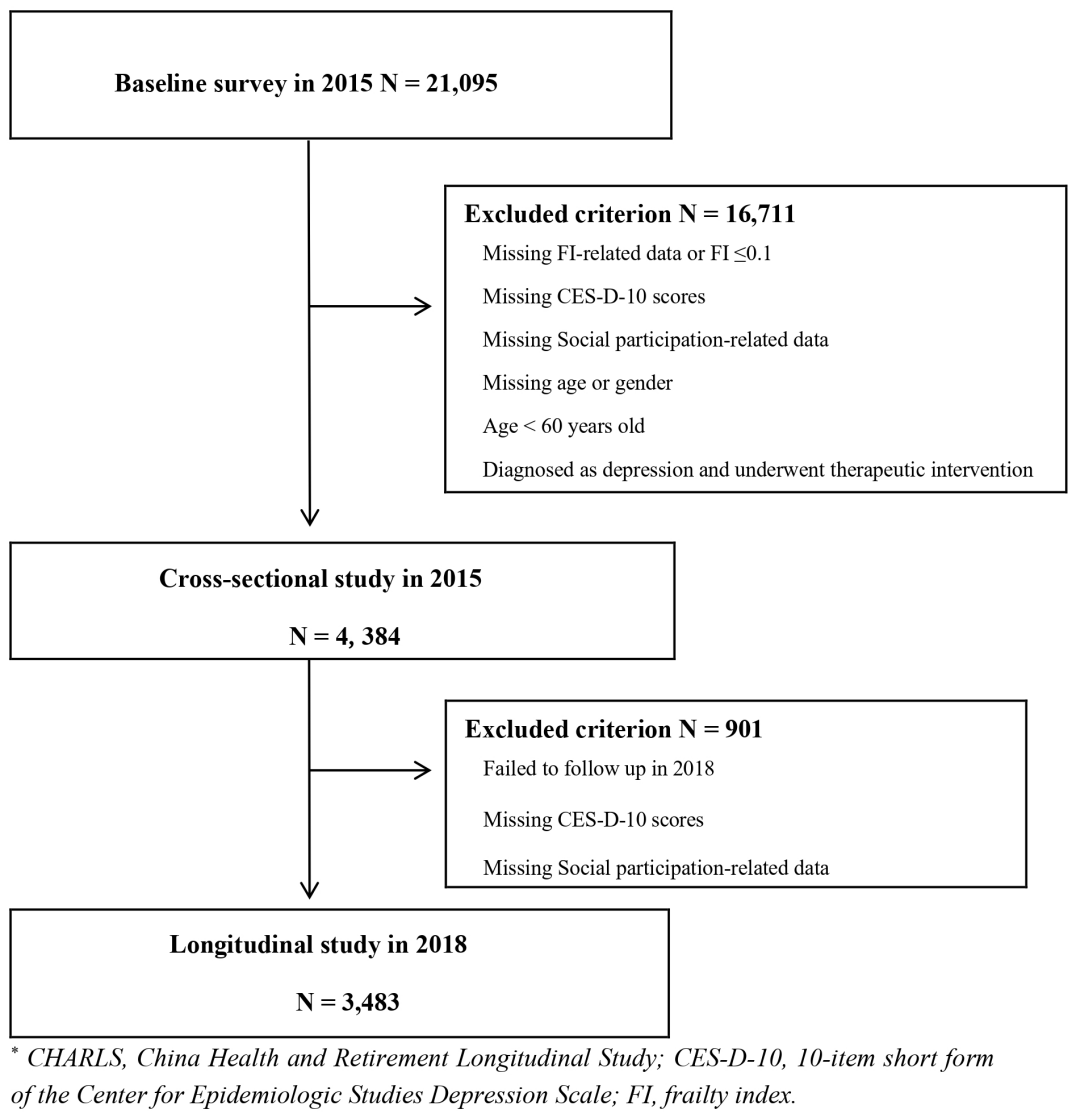
Flowchart of total study participant involvement.

Our study used wave 3 (December 2015 to January 2016) and wave 4 (July to September 2018) data from CHARLS. The final analysis involved 4,384 participants, using the 2015 survey data as the baseline for comparison throughout the study. A total of 901 participants were excluded due to missing important information (e.g., age and gender), incomplete depression questionnaires, loss to follow-up, death, or failure to engage in the depression questionnaire. The number of respondents included in the 2018 follow-up study was 3,483 in total. **Figure 1** shows the entire research participation process.

### 2.2 Outcome measures

#### 2.2.1 Assessment of frailty

For the purpose of assessing frailty, the frailty index (FI) was utilized. According to prior research, the frailty index (FI) has a strong predictive capacity for adverse clinical outcomes and may better represent the general health state of the geriatric population (34, 35). The FI was calculated in accordance with the standards for the deficit accumulation model that were developed by the Searle team (36). In constructing the FI, forty items were selected; these included self-reported health status, disability, comorbidities, functional limitations, depressive symptoms, and cognitive status. The specific items of FI are displayed in **Supplementary Table 1**. The FI is computed by dividing the sum of all scores (from 0 to 40) by 40, according to the formula. According to Roman and Rose, an FI of 1 signifies inadequate performance across all domains, whereas a value of 0 indicates the absence of any indications or symptoms of frailty (37). Frailty status in this research encompasses both prefrailty and frailty participants, who are characterized by an FI value > 0.1 (38).

#### 2.2.2 Assessment of social participation

Social participation behaviors were defined in the CHARLS questionnaire as those in which the participants were involved in the previous month, among the ten activities that involved visiting relatives and friends, chess and mahjong, helping relatives or neighbors, and sports activities (such as dancing, fitness, Qigong, etc.), participating in volunteers or activities, caring for patients or disabled people, training classes, stock trading, and surfing the Internet. This research calculated the number of social behavior types among the participants and employed latent class analysis to investigate social participation patterns.

#### 2.2.3 Assessment of severity of depressive symptoms

Depression level was identified using the 10-item short form of the Center for Epidemiologic Studies Depression Scale (CES-D-10). With strong reliability and validity in the Chinese aged population, the CES-D-10 is a frequently used instrument in large-sample epidemiological studies to assess depressive symptoms (39, 40). For each symptom in the previous week, the CES-D-10 assigns a total of 30 points to each symptom, ranging from 0 (indicating little or no occurrence [< 1 day]) to 3 (indicating most or all occurrence [5-7 days]); greater scores indicate more severe depressive symptoms. The specific problems are shown in **Supplementary Table 2**. Reverse scoring was applied to the two positive emotion questions “I feel hopeful about the future” and “I was happy” when calculating the total scores from the CES-D-10 questionnaire divided the participants into three groups: those with depressive disorder (DD) (scores equal to or exceeding 20), those with depressive symptoms (DS) (scores equal to or exceeding 10), and those with non-depressive symptoms (NDS) (scores below 10) (41, 42). Furthermore, we regard an increase in the severity of depression among the participants from 2015 to 2018 as an indication that their depressive symptoms were deteriorating.

### 2.3 Other covariates

At the baseline survey, relevant confounding variables such as demographics, behavioral traits, and other aspects were gathered. The main factors included age, gender, residence (rural, urban), marital status (married, separated/divorced/widowed, unmarried), education degree (illiteracy, elementary school/middle school, high school/higher education), family annual total income (<10,000 yuan, 10,000-50,000 yuan, 50,000-100,000 yuan, >100,000 yuan), smoking status (never smoked, continued smoking, quit smoking), drinking status (with or without drinking habits), total sleep duration and body mass index (BMI).

### 2.4 Statistical analysis

All statistical analyses were conducted by MPlus7.4 and R4.2.2. A two-sided p value < 0.05 was considered statistically significant in this study. Categorical variables are represented as percentages (absolute values), while continuous variables are labelled as the mean ± (standard deviation, SD). To compare differences in categorical variables between groups, the χ2 test or Wilcoxon rank sum test was utilized. To examine the differences in continuous variables between groups, Student’s t test, Kruskal−Wallis test, or analysis of variance were utilized.

Utilizing response surface analysis, the congruence effect between the number of social types and FI with respect to symptoms of depression was examined. By employing regression coefficients, we generated three-dimensional response surfaces. The vertical axis of these surfaces represented the CES-D-10 scores (Z), while the perpendicular horizontal axes represented FI (X) and the number of social participation types (Y). As a result of R2’s significance, the differences in the participants’ depressive symptoms can be substantially accounted for. The congruence effect between the number of social types and FI on depressive symptoms is indicated by the significance of the curvature of the incongruence line (P=-A) (43).

Latent Class Analysis (LCA) is a statistical technique designed to identify mutually exclusive groups of respondents, each characterized by its own unique set of probabilities for endorsing symptoms (44, 45). The goal of LCA is to determine an optimal classification scheme where each group is as homogeneous as possible internally, while being as distinct as possible from other groups. Using LCA, the social participation patterns of older adults in 2015 and 2018 were classified separately. To determine the most suitable latent class model, various criteria were used for comparison. Specifically, the Akaike Information Criterion (AIC), Bayesian Information Criterion (BIC), and sample size adjusted BIC (aBIC) were considered, with lower values indicating a better model fit. Entropy was used to assess the clarity of the latent classes, with values above 0.8 being considered excellent. Additionally, the Bootstrap Likelihood Ratio Test (BLRT) was used to determine if adding more classes significantly improves the model, with a p-value less than 0.05 indicating significance. Together, these criteria—lower AIC, BIC, and aBIC values, higher entropy, and a significant bootstrap likelihood ratio test—guide the selection of the optimal number of latent classes (46, 47).

Following the classification of the data, significant covariates were incorporated into the regression mixture model (RMM) using the straightforward three-step most likely class regression approach to ascertain their potential contribution to the latent classification. By using depression levels as the outcome variable in RMM, we may explore how latent classes affect depression levels. Then, the study employed multiple variables to construct three ordered logistic regression models that investigate the impact of social latent classes on depression. Model 1 adjusts solely for age and gender, Model 2 for all demographic variables, and Model 3 for demographic and behavioral variables.

We used Tihomir Asparouhov’s three-step modeling technique to perform a latent transition analysis (LTA) of social participation patterns from 2015 to 2018. First, as previously stated, we completed the 2015 and 2018 latent classification of social participation. The second step is to prove measurement consistency in the latent classes of participation patterns in both years. The two models’ fitting indicators were assessed using the log-likelihood ratio test (LRT) with equal parameter changes across time. Then, the transition matrix obtained from 2015 to 2018 of the potential classification by LTA. This analysis allowed us to discern the stability and transition rates of each latent class. We then used the findings from LTA as independent variables and worsening depression symptoms as dependent variables in three ordered logistic regression models. This approach aims to evaluate how different transition patterns in social participation relate to depression levels, providing a deeper understanding of how social involvement impacts mental health.

## 3 Results

### 3.1 Demographic characteristics and depression severity

Our cross-sectional study comprised 4,384 individuals, with 901 excluded due to reasons such as incomplete data and loss of follow-up, resulting in 3,483 respondents in the longitudinal cohort three years later. The baseline survey showed an average age of 68.71 years (SD = 6.74). Participants were 58.53% female (2,566) and 41.47% male (1,818). A majority, 67.85% (2,746), lived in rural areas, while 32.15% (1,305) were urban residents. Marital status was 54.30% married or cohabitating (2,221), 29.50% single (1,218), and 16.20% divorced or widowed (674). Other demographic, behavioral, and risk factor variables are shown in **Table 1**. **Table 1** shows that participants who were younger, female, lived in rural settings, were illiterate, reported lower household incomes, had shorter sleep durations, and exhibited lower BMI demonstrated elevated levels of depression severity. Furthermore, our analysis identified that participants in the DD group and the DS group had significantly higher FI and fewer types of social participation compared to NDS group.

**Table 1:** Baseline characteristics of participants by depressive symptom status (2015, N = 4,384).

Characteristic		Total N = 4,384	Depression symptom			P-value
			*NDS group	*DS group	*DD group	
Age		68.71 (6.74)	69.09 (7.06)	68.54 (6.56)	67.81 (6.05)	0.012
Gender	Man	41.47 (1,818)	47.02 (876)	38.82 (825)	29.55 (117)	< 0.001
	Women	58.53 (2,566)	52.98 (987)	61.18 (1,300)	70.45 (279)	
Residence	Urban	25.34 (1,108)	29.71 (552)	23.28 (493)	15.91 (63)	< 0.001
	Rural	74.66 (3,264)	70.29 (1,306)	76.72 (1,625)	84.09 (333)	
Marital status	Married/cohabitating	76.46 (3,352)	79.17 (1,475)	75.11 (1,596)	70.96 (281)	0.001
	Divorced/separated/widowed	22.72 (996)	20.34 (379)	23.91 (508)	27.53 (109)	
	Never married	0.82 (36)	0.48 (9)	0.99 (21)	1.52 (6)	
Education degree	Illiteracy	43.93 (799)	43.84 (345)	42.53 (370)	51.85 (84)	0.007
	Elementary school/middle school	54.40 (935)	49.81 (392)	54.02 (470)	45.06 (73)	
	High school/higher education	4.67 (85)	6.35 (50)	3.45 (30)	3.09 (5)	
Total annual household income	<10,000 yuan	34.01 (1,491)	30.06 (560)	36.05 (766)	41.67 (165)	< 0.001
	10,000-50,000 yuan	22.08 (968)	23.19 (432)	21.41 (455)	20.45 (81)	
	50,000-100,000 yuan	5.57 (244)	7.14 (133)	4.80 (102)	2.27 (9)	
	>100,000 yuan	38.34 (1,681)	39.61 (738)	37.74 (802)	35.61 (141)	
Smoke status	Never smoked	61.23 (2,682)	59.00 (1,098)	62.24 (1,322)	66.33 (262)	0.010
	Continued smoking	15.07 (660)	16.87 (314)	14.27 (303)	10.89 (43)	
	Quit smoking	23.70(1,038)	24.13 (449)	23.49 (499)	22.78 (90)	
Drink habit	Yes	27.24 (1,193)	29.50 (549)	26.33 (559)	21.46 (85)	0.002
Total sleep duration		6.67 (2.48)	7.22 (2.34)	6.38 (2.48)	5.65 (2.59)	< 0.001
*BMI		23.55 (4.12)	23.77 (4.07)	23.40 (4.08)	23.28 (4.55)	< 0.001
*CES-D-10 scores		10.97 (6.36)	5.04 (2.65)	13.89 (2.83)	23.29 (2.80)	< 0.001
*FI		0.26 (0.13)	0.22 (0.10)	0.29 (0.12)	0.38 (0.15)	< 0.001
Number of social participation types		0.73 (0.97)	0.82 (1.03)	0.69 (0.94)	0.57 (0.84)	< 0.001

^*^Variables are presented as percentages (number), or mean (SD, standard deviation).

^*^NDS, non-depressive symptom; DS, depressive symptom; DD, depressive disorder; BMI, body mass index; CES-D-10, 10-item short form of the Center for Epidemiologic Studies Depression Scale; FI, frailty index.

^*^P-value less than 0.05 was defined as significant.

### 3.2 Latent class of social participation pattern

Social participation patterns were classified using multi-LCA models. From 2 to 7 subgroups or classes were examined. Based on the lowest AIC, BIC, and Adjusted BIC values, high entropy above 0.9, and significant BLRT, the 5-class model was identified as the most suitable. From a clinical standpoint, the five latent classes are distinctly defined and exhibit unique characteristics, rendering them both clinically relevant and interpretable. Each class presents specific attributes that provide valuable insights for clinical practice and intervention. Fit indexes for the final 5-class model were AIC=17682.79; BIC=18059.55; Adjusted BIC=17872.07; Entropy=0.95. Fit indexes for 7 disaggregated models are presented in **Supplementary Table 3**. After considering model fit indexes, cluster size, and practical clinical significance, a 5-class solution best represented the data. Based on the LCA result of participants’ social activity engagement patterns, we identified five distinct categories: Class 1 “socially isolated”, Class 2 “socialize with friends”, Class 3 “board game enthusiast”, Class 4 “helpful individual”, and Class 5 “extensive social interaction”, as illustrated in **Supplementary Figure 1**. Participants classified as Class 1 “socially isolated” engage in very few social activities. Class 2 participants “socialize with friends” frequently interact with friends but are less involved in other social activities. Class 3, “board game enthusiasts”, primarily engage in social activities through board games such as poker, mahjong, etc. Class 4, the “helpful individuals”, are characterized by active participation in volunteer work or other helping activities. Lastly, Class 5 “extensive social interaction” participants participate in a broad range of social and interest-related activities, reflecting a high level of social interaction. In the baseline survey, 52.78% of frail older adults belonged to Class 1. Class 2, Class 3, Class 4, and Class 5 comprised the remaining 24.34%, 6.14%, 11.31%, and 5.43%, respectively. **Supplementary Table 4** displays the specific distribution of various latent social participation classes in 2015. As observed in the table, individuals in Class 1 had an extremely low likelihood (≤ 0.001) of engaging in any aspect of social activity participation. Similarly, those in Class 2, Class 4 and Class 3 had a higher probability of corresponding items. People in Class 5 had a higher probability in most of the activity items than others. In our LCA analysis for the year 2018, we discovered comparable findings (**Supplementary Table 5**).

### 3.3 Differences among the social participation patterns

A comparative overview of the baseline characteristics of five latent social participation classes in 2015 is presented in **Table 2**. There is substantial variation across the five classes in terms of age, gender, marital status, place of residence, drinking habits, smoking status, total annual household income, BMI, FI, and CES-D-10 scores. However, the total sleep duration did not show significant differences among the classes (p > 0.05). More specifically, Class 1 is distinguished by a larger proportion of rural residents, older individuals, those with lower household incomes, and those with higher FI. Conversely, Class 5 is primarily composed of older people with higher BMI. Furthermore, we noted that Class 2 predominantly comprises younger individuals with higher household incomes, whereas Class 3 is associated with individuals who have lower BMI and CES-D-10 scores. On the other hand, Class 4 is distinguished by a more balanced distribution of socioeconomic and health-related characteristics.

**Table 2:** Characteristics of participants by various latent social participation classes in 2015 (N = 4,384).

Characteristic		Total N = 4,384	Social participation pattern					P-value
			Class 1 Socially isolated	Class 2 Socialize with friends	Class 3 Board game enthusiast	Class 4 Helpful individual	Class 5 Extensive social interaction	
Age		68.70 (6.74)	69.03 (6.82)	69.01 (6.91)	68.34 (6.69)	67.35 (5.98)	67.01 (6.13)	< 0.001
Gender	Man	41.47 (1,818)	41.70 (965)	36.46 (389)	57.25 (154)	46.37 (230)	33.61 (80)	< 0.001
	Women	58.53 (2,566)	58.30 (1,349)	63.54 (678)	42.75 (115)	53.63 (266)	66.39 (158)	
Residence	Urban	25.34 (1,108)	21.62 (499)	23.24 (247)	37.17 (100)	25.25 (125)	57.81 (137)	< 0.001
	Rural	74.66 (3,264)	78.38 (1,809)	76.76 (816)	62.83 (169)	74.25 (370)	42.19 (100)	
Marital status	Married/cohabitating	76.46 (3,352)	77.87 (1,802)	71.79 (766)	78.07 (210)	79.44 (394)	75.63 (180)	0.013
	Divorced/separated/widowed	22.72 (996)	21.26 (492)	27.46 (293)	21.19 (57)	19.76 (98)	23.53 (56)	
	Never married	0.82 (36)	0.86 (20)	0.75 (8)	0.74 (2)	0.81 (4)	0.84 (2)	
Education degree	Illiteracy	43.93 (799)	49.85 (483)	42.92 (188)	22.88 (27)	36.32 (77)	29.27 (24)	< 0.001
	Elementary school/middle school	54.40 (935)	46.75 (453)	52.97 (232)	71.17 (84)	55.66 (118)	58.54 (48)	
	High school/higher education	4.67 (85)	3.41 (33)	4.11 (18)	5.93 (7)	8.02 (17)	12.20 (10)	
Total annual household income	<10,000 yuan	34.01 (1,491)	36.73 (850)	34.11 (364)	27.14 (73)	32.46 (161)	18.07 (43)	< 0.001
	10,000-50,000 yuan	22.08 (968)	21.43 (496)	23.52 (251)	21.56 (58)	20.77 (103)	25.21 (60)	
	50,000-100,000 yuan	5.57 (244)	5.06 (117)	5.44 (58)	6.69 (18)	6.85 (34)	7.14 (17)	
	>100,000 yuan	38.34 (1,687)	36.78 (851)	36.93 (394)	44.61 (120)	39.92 (198)	49.58 (118)	
Smoke status	Never smoked	61.23 (2,682)	62.15 (1,437)	63.32 (675)	46.84 (126)	57.17 (283)	67.65 (161)	< 0.001
	Continued smoking	15.07 (660)	15.35 (355)	13.32 (142)	18.22 (49)	15.15 (75)	16.39 (39)	
	Quit smoking	23.70 (1,038)	22.49 (520)	23.36 (249)	34.94 (94)	27.68 (137)	15.97 (38)	
Drink habit	Yes	27.24 (1,193)	24.51 (567)	26.85 (286)	32.09 (86)	37.70 (187)	28.15 (67)	< 0.001
Total sleep duration		6.67 (2.48)	6.65 (2.55)	6.66 (2.45)	6.62 (2.22)	6.88 (2.52)	6.47 (2.22)	0.081
*BMI		23.55 (4.12)	23.36 (4.09)	23.8 (4.10)	23.68 (3.54)	23.34 (4.37)	24.47 (4.54)	< 0.001
*FI		0.26 (0.13)	0.28 (0.14)	0.26 (0.12)	0.23 (0.10)	0.24 (0.11)	0.23 (0.10)	< 0.001
Depression symptom	*NDS group	42.50 (1,863)	39.54 (915)	44.05 (470)	53.16 (143)	43.15 (214)	50.84 (121)	< 0.001
	*DS group	48.47 (2,125)	50.09 (1,159)	47.33 (505)	41.26 (111)	48.79 (242)	45.38 (108)	
	*DD group	9.03 (396)	10.37 (240)	8.62 (92)	5.58 (15)	8.06 (40)	3.78 (9)	
*CES-D-10 scores		10.97 (6.36)	11.45 (6.48)	10.79 (6.29)	9.60 (5.89)	10.62 (6.21)	9.41 (5.72)	< 0.001

^*^Variables are presented as percentages (number), or mean (SD, standard deviation).

^*^BMI, body mass index; CES-D-10, 10-item short form of the Center for Epidemiologic Studies Depression Scale; FI, frailty index; NDS, non-depressive symptom; DS, depressive symptom; DD, depressive disorder.

^*^P-value less than 0.05 was defined as significant.

By including age, gender, and FI as predictor variables in the RMM, the results demonstrate that these factors substantially contribute to the classification of social participation patterns. Taking Class 1 as the reference, age had negative regression coefficients for Class 5, Class 4, and Class 3, with values of -0.055, -0.039, and -0.015, respectively (P < 0.05) (**Supplementary Table 6**). This implies that an increase in age reduces the likelihood of individuals belonging to Class 3, Class 4, and Class 5 in comparison to Class 1. To be more precise, the relative probabilities of belonging to Class 3, Class 4, and Class 5 decrease by 0.015, 0.039, and 0.055 for each one-unit increase in age, respectively. In the same way, all four subgroups demonstrated negative regression coefficients for the frailty index. In addition, the regression coefficients for females in Class 5 and Class 2 are positive.

### 3.4 Associations between social participation patterns and depression degree

**Supplementary Table 7** presents the outcomes obtained by including depression severity as the outcome variable in RMM. The results indicate that in Class 1, the probabilities of belonging to this class were significantly higher for individuals in the DS group (0.395) and DD group (0.501) compared to those in other classes. Conversely, frail older adults in Class 5 (0.527) and Class 3 (0.520) exhibited notably higher probabilities of being in the NDS group in 2015 than those in other classes. These findings indicate that the severity of depression influences latent class membership. Individuals with more severe depression are more likely to belong to the DS and DD groups in Class 1. In contrast, individuals with less depression symptoms are more likely to be in the NDS group in Classes 5 and 3.

The results of the ordered regression analysis in 2015 are presented in **Table 3**, which illustrates the correlation between various latent classes and the severity of depression symptoms. Confounding variables were accounted for in three of the models. The findings of our analysis suggest that, upon controlling for all pertinent variables (model 3), the probability of experiencing a one-level escalation in depression symptoms is lower for all other subgroups compared to Class 1. Specifically, individuals in Class 5 had a 33% lower likelihood (95% CI, 0.49-0.90; P < 0.01) of experiencing a one-level increase in depression symptoms compared to those in Class 1, while individuals in Class 3 had a 38% lower likelihood (95% CI, 0.47-0.82; P < 0.01). These results indicate that, relative to Class 1, older adults in Classes 5 and 3 have a reduced risk of experiencing an escalation in depression symptoms. Similar outcomes were also observed in the ordered logistic regression findings of 2018 (**Table 4**).

**Table 3:** Associations between different latent social participation classes and depression degree in 2015.

	Model 1^a^	Model 2^b^	Model 3^c^
	*OR (95% *CI)	OR (95% CI)	OR (95% CI)
Class 1	Ref	Ref	Ref
Class 2	0.83 (0.72-0.95)^†^	0.83 (0.71-0.97)^†^	0.83 (0.71-0.97)^†^
Class 3	0.57 (0.44-0.73)^‡^	0.62 (0.47-0.82)^‡^	0.62 (0.47-0.82)^‡^
Class 4	0.84 (0.70-1.02)	0.84 (0.68-1.03)	0.83 (0.68-1.03)
Class 5	0.60 (0.46-0.77)^‡^	0.66 (0.49-0.89)^‡^	0.67 (0.49-0.90)^‡^

a Adjusted for age and gender.

b Adjusted for age, gender and other demographic variables (including residence, marital status, family annual total income).

c Adjusted for all demographic and health behavioral variables (including BMI, smoking status, drink habit, FI, number of social participation types).

^*^OR, odds ratio; CI, confidence intervals; FI, frailty index.

^‡^p < 0.01, ^†^p < 0.05.

**Table 4:** Associations between different latent social participation classes and depression degree in 2018.

	Model 1^a^	Model 2^b^	Model 3^c^
	*OR (95% *CI)	OR (95% CI)	OR (95% CI)
Class 1	Ref	Ref	Ref
Class 2	0.83 (0.71-0.98)^†^	0.84 (0.71-0.98)^†^	0.85 (0.72-0.99)^†^
Class 3	0.64 (0.46-0.78)^‡^	0.66 (0.50-0.87)^‡^	0.66 (0.50-0.88)^‡^
Class 4	0.93 (0.75-1.21)	0.93 (0.73-1.18)	0.92 (0.72-1.17)
Class 5	0.52 (0.41-0.67)^‡^	0.57 (0.44-0.73)^‡^	0.57 (0.45-0.73)^‡^

a Adjusted for age and gender.

b Adjusted for age, gender and other demographic variables (including residence, marital status, family annual total income).

c Adjusted for all demographic and health behavioral variables (including BMI, smoking status, drink habit, FI, number of social participation types).

^*^OR, odds ratio; CI, confidence intervals; FI, frailty index.

^‡^p < 0.01, ^†^p < 0.05.

### 3.5 Latent transition of social participation patterns from 2015 to 2018

**Figure 2:**
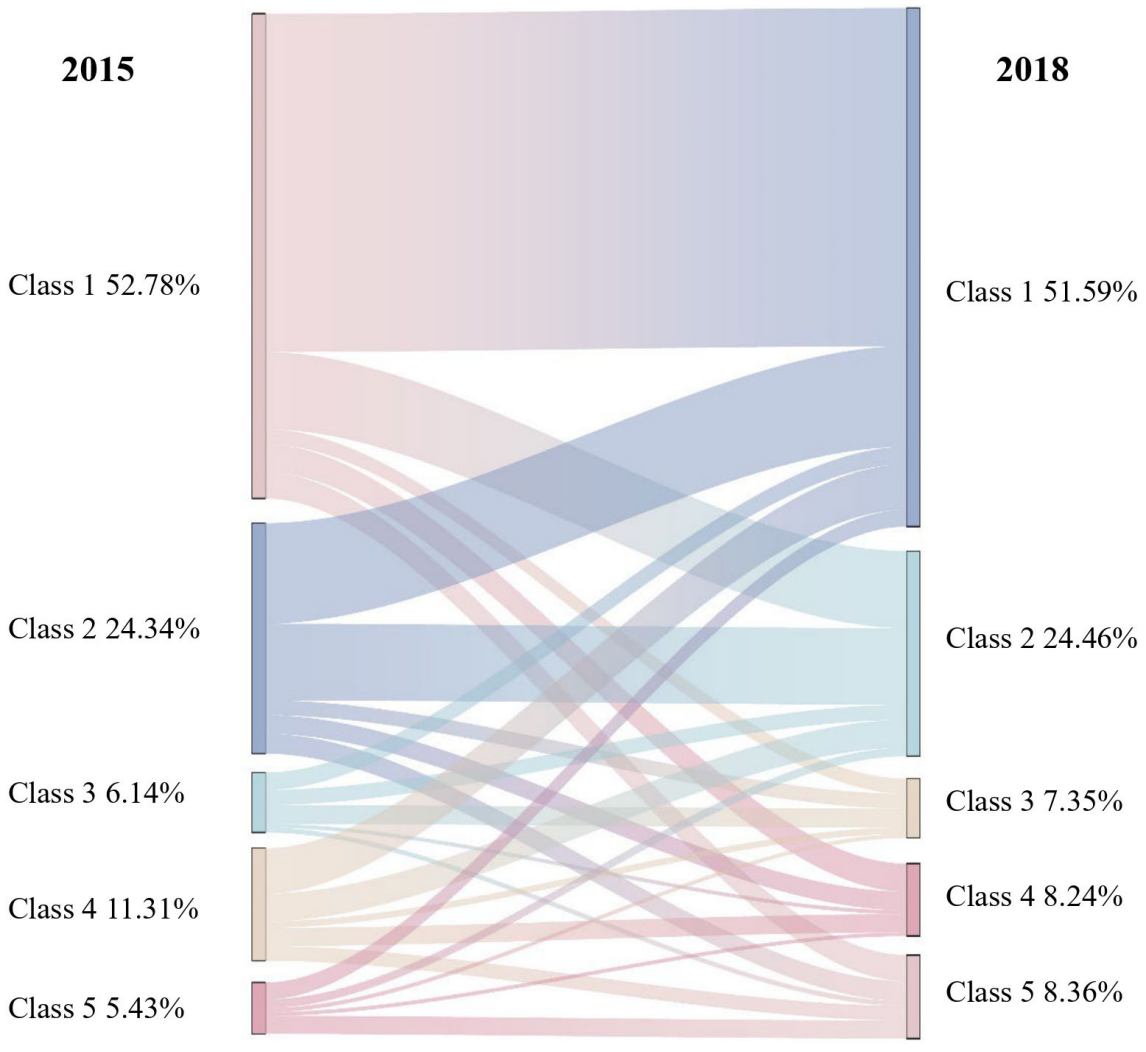
Result of transition probabilities from 2015 to 2018.

At two time points—from 2015 to 2018—LTA was utilized to examine the longitudinal transition of latent social participation classes. In this investigation, 3,483 participants were included in total. Our findings indicate that the majority of participants maintained a consistent pattern of social participation during the follow-up period (**Figure 2**). However, a smaller proportion of people modified their social participation behavior. Class 1 was the most stable, with a 0.85 likelihood of remaining unaltered from 2015 to 2018. Class 2 had a probability of remaining unchanged of 0.585. The probability of Class 5 members transitioning to an alternative class in 2015 was as great as 0.738 percent. One of these individuals had a 0.704% likelihood of advancing to Class 2 in 2018.

Furthermore, according to the findings of logistic regression analysis, there was a greater likelihood of depressive symptoms worsening among individuals who transitioned from Class 1 to Class 4 (OR, 1.56; 95% CI, 1.00-2.44; P = 0.050) in 2018 as opposed to those who remained in Class 1. Meanwhile, as older adults shift from Class 4 to Class 1, the probability of experiencing a deterioration in their levels of depression decreases. However, this finding does not show statistical significance. Conversely, there was a markedly reduced likelihood of depressive symptom worsening among individuals who changed from Class 1 to Class 3 (OR, 0.20; 95% CI, 0.62-0.65; P = 0.008). Similar results were observed in individuals transitioning from the Class 1 to the Class 5 subgroup, but this result was not statistically significant. At the same time, we found that transitioning in the opposite direction led to a significantly decreased probability of worsening depression levels (Class 3 to Class 1: OR, 2.35; 95% CI, 1.01-5.48; P = 0.047; Class 5 to Class 1: OR, 2.51; 95% CI, 1.18-5.32; P = 0.017). The results of the aforementioned logistic regression with respect to the transition are presented in **Table 5**.

**Table 5:** Association between depressive symptom deterioration and different transition pattern in social participation classes from 2015 to 2018.

Transition pattern	deteriorate	P
Class 1 → Class 1 (no transition)	Ref	
Class 1 → Class 2	1.21 (0.89-1.63)	0.220
Class 1 → Class 3	0.20 (0.62-0.65)	0.008
Class 1 → Class 4	1.56 (1.00-2.44)	0.050
Class 1 → Class 5	0.96 (0.57-1.60)	0.866
Class 2 → Class 2 (no transition)	Ref	
Class 2 → Class 1	0.91 (0.61-1.34)	0.624
Class 2 → Class 3	0.71 (0.31-1.61)	0.412
Class 2 → Class 4	0.82 (0.41-1.64)	0.575
Class 2 → Class 5	0.74 (0.37-1.49)	0.404
Class 3 → Class 3 (no transition)	Ref	
Class 3 → Class 1	2.35 (1.01-5.48)	0.047
Class 3 → Class 1	1.95 (0.80-4.74)	0.141
Class 3 → Class 4	1.47 (1.05-4.00)	0.487
Class 3 → Class 5	1.00 (0.23-4.20)	0.997
Class 4 → Class 4 (no transition)	Ref	
Class 4 → Class 1	0.82 (0.27-2.46)	0.726
Class 4 → Class 2	1.59 (0.51-4.97)	0.421
Class 4 → Class 3	2.00 (0.38-1.53)	0.413
Class 4 → Class 5	0.97 (0.23-4.07)	0.962
Class 5 → Class 5 (no transition)	Ref	
Class 5 → Class 1	2.51 (1.18-5.32)	0.017
Class 5 → Class 2	1.12 (0.45-2.80)	0.805
Class 5 → Class 3	0.78 (0.22-2.82)	0.707
Class 5 → Class 4	1.97 (0.60-6.50)	0.267

### 3.6 Congruence and incongruence effects on depressive symptoms

**Figure 3:**
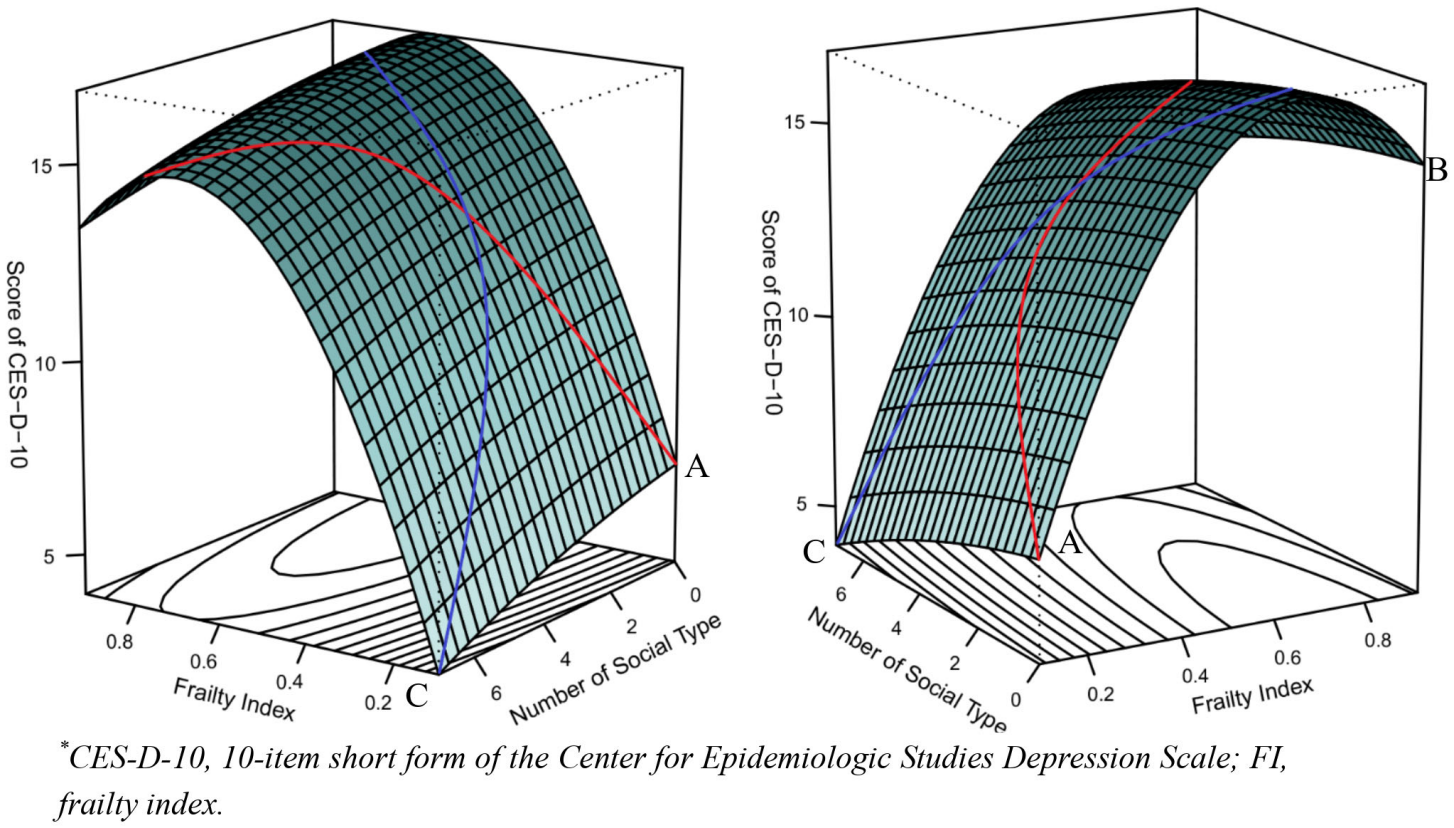
Congruence and incongruence effects of FI and the number of social participation type on CES-D-10 scores.

Two perspectives of the results of the response surface analysis are illustrated in **Figure 3**. The analysis shows that, along the incongruence line (X = -Y, represented by the blue line), the CES-D-10 score was significantly lower at point A (low FI, higher number of social types) compared to point B (high FI, lower number of social types). In contrast, along the congruence line (X = Y, represented by the red line), the CES-D-10 score at point C (low FI, higher number of social types) was notably lower than at point A. Overall, these findings suggest that a higher number of social types is associated with reduced depressive symptoms, and this positive effect of social engagement outweighs the negative impact of FI.

## 4 Discussion

By employing a representative nationwide sample from the CHARLS longitudinal cohort study, this study thoroughly investigated the correlation between frailty, social participation and depression symptoms. In our study, different social participation classes have varying effects on depression symptoms in frail older adults. We also noticed that the transitions in social classes during the follow-up period exerted distinct influences on the depression symptoms among these individuals. Moreover, the variety of social participation and frailty status may present opposite trends in terms of the impact on depression symptoms.

Older female adults in rural areas, who are single or not cohabiting, have lower income, shorter sleep duration, and lighter weight, appear to have more severe depressive symptoms according to the baseline survey. These results are consistent with previous research (48, 49). Older individuals who are single or do not live with a partner are more vulnerable to social isolation and loneliness. Their limited access to essential social support and emotional dependence in daily life may increase the likelihood of developing depressive symptoms (50, 51). Individuals with low income frequently encounter heightened financial adversity and life stress, which may result in a sense of helplessness and despair, ultimately elevating the risk of developing depression. Numerous studies have shown how unstable financial status negatively impacts psychological health, and diverse populations have extensively verified these effects (52–54). Moreover, there is a bidirectional connection between depression and both sleep quality and sleep duration (55, 56). This not only reflects that reduced sleep duration and poor sleep quality are symptoms of depression but also indicates that sleep can influence the onset and progression of depressive symptoms.

It is worth noting in this study that older people having a higher frailty index and engaging in fewer types of social activities may contribute to an increase in depressive symptoms. According to the findings of a study conducted by Patrick J. in 2019, there is a connection between frailty, specifically physical weakness and reduced mobility and strength, and more severe depressive symptoms (57). Physical and mental tasks that require substantial energy are often beyond the reach of frail older adults. As a result, they may struggle with feelings of helplessness and diminished self-worth, and find it challenging to handle their daily affairs. This limitation also restricts their ability to participate in social activities, hence decreasing their opportunities for interaction with others to a certain degree. The decrease in social engagement may exacerbate depression symptoms in older people and further deteriorate their mental and physical health. Furthermore, Zhu and his colleagues used a Mendelian randomization strategy to reveal a genetically based bidirectional causal link between frailty and depression (58). The results of this study indicate that frailty is not only a risk factor for depression, but that depression may also exacerbate the severity of frailty.

This study used the LCA method to classify the social engagement patterns of frail older adults into five latent classes: “socially isolated”, “socialize with friends”, “board game enthusiasts”, “helpful individuals” and “extensive social interaction”. According to the findings, those with higher FI are more likely to be classified as members of class 1, referred to as “socially isolated”. This may offer an explanation as to why class 1 had the largest proportion of individuals in both 2015 and 2018, since our study focused exclusively on frail older adults. A research study on frailty and social relationship types among elderly in 17 European countries reported similar results to ours (59). The corresponding result in our study was that the “extensive social interaction” subclass and the “socially isolated” subgroup had completely opposing effects on the likelihood of worsening depressive symptoms. Interestingly, according to the results of RMM and ordered logistic regression, the “board game enthusiast” subgroup significantly reduces the likelihood of the incidence and worsening of depression symptoms. Playing these games as part of daily social activities may provide cognitive stimulation and challenge, assist in shifting attention, changing negative thought habits, and increasing pleasant emotional experiences, all of which promote mental health. In light of this discovery, additional research could investigate the mechanisms through which board games influence depressive symptoms, with the aim of reaching a more comprehensive comprehension of their implications for the mental well-being of frail older adults. This result aligns with the findings in previous research (60). This study revealed that urban inhabitants experienced a beneficial impact on their mental well-being through engagement in these activities, while their rural counterparts did not demonstrate comparable outcomes. This difference may be attributed to variations in the social environment, cultural background, and lifestyle between urban and rural residents.

We identified some interesting trends in the logistic regression results of potential transitions from 2015 to 2018. More specifically, individuals transitioning from Class 1 to Class 4 in 2018 had a higher probability of worsening depressive symptoms than those who remained in Class 1. However, when transitioning from Class 4 to Class 1, the likelihood of worsening depressive symptoms decreased. This may mean that individuals in Class 1, who have less social interaction, may face more psychological stress when transitioning to Class 4 with more social interaction, leading to a worsening of depressive symptoms. Related studies suggest that a long-term social pressure may contribute to the development of depression in individuals (61). This could potentially be attributed to the decreased expression of neural stability-related proteins in individuals under prolonged social pressure, leading to the occurrence of depressive disorder (62, 63).

The relationship among FI, the number of social activity types, and the severity of depression, as analyzed by RSM in our study. When both the FI and the number of social activity types increase, it indicates that the individual has a higher level of frailty and engages in a wider range of social activities. The findings suggest that this combination may result in higher levels of depression symptoms. On the other hand, when there is a decrease in FI and an increase in the types of social activities, the situation seems to be more favorable. This implies that the potential benefits of having a diverse range of social types may mitigate in part the adverse effects of frailty on symptoms of depression, especially for individuals with lower levels of frailty.

### 4.1 Limitations

There are several limitations to this study that must be taken into account. In the present investigation, patients’ frailty status was quantified utilizing the FI, while symptoms of depression were evaluated employing the CES-D-10 scores. Nonetheless, it is important to note that the CES-D-10 scores and the remaining 39 items of data gathered for the FI were all self-reported by the participants. This approach introduces the possibility of subjectivity and the potential for inaccurate or biased results. Consequently, it would be prudent to use a variety of assessment instruments or incorporate clinical diagnoses for validation in future studies. Second, the CHARLS questionnaire only records individuals’ social activity participation in the previous month. This approach may aim to minimize the impact of long-term memory on participants. Due to its focus on survey data from the month prior, it may not accurately reflect participants’ long-term social activity. Future research could benefit from an expanded time frame or data from multiple time periods in the survey to obtain a more comprehensive understanding of the social activity participation of the participants.

## 5 Conclusions

This cohort study found the evidence of the association between frailty, social participation, and depression symptoms. Different types of social participation and transitions between social participation patterns may significantly impact depressive symptoms in frail older adults. Furthermore, this study also found that social participation type number have a greater impact on depression levels compared to frailty status. These results provide guidance on addressing and supporting the mental health of older adults. Further research is needed to better understand how depressive symptoms impact frail older adults and recommend more specific interventions for effective interventions.

## Data Availability

The original contributions presented in the study are included in the article/**Supplementary Material**. Further inquiries can be directed to the corresponding authors.

## References

[1] GBD 2017 Diet Collaborators. Health effects of dietary risks in 195 countries, 1990-2017: a systematic analysis for the Global Burden of Disease Study 2017. (2019). doi: 10.1016/S0140-6736(19)30041-8 PMC6899507 30954305

[2] Thillainadesan J Scott IA Le Couteur DG. Frailty, a multisystem ageing syndrome. Age Ageing. (2020) 49:758–63. doi: 10.1093/ageing/afaa112 32542377

[3] O’Caoimh R Sezgin D O’Donovan MR Molloy DW Clegg A Rockwood K. Prevalence of frailty in 62 countries across the world: A systematic review and meta-analysis of population-level studies. Age Ageing. (2021) 50:96–104. doi: 10.1093/ageing/afaa219 33068107

[4] Muntsant A Giménez-Llort L. Crosstalk of Alzheimer’s disease-phenotype, HPA axis, splenic oxidative stress and frailty in late-stages of dementia, with special concerns on the effects of social isolation: A translational neuroscience approach. Front Aging Neurosci. (2022) 14:969381. doi: 10.3389/fnagi.2022.969381 36185472 PMC9520301

[5] Soysal P Veronese N Thompson T Kahl KG Fernandes BS Prina AM. Relationship between depression and frailty in older adults: A systematic review and meta-analysis. Ageing Res Rev. (2017) 36:78–87. doi: 10.1016/j.arr.2017.03.005 28366616

[6] Dugravot A Fayosse A Dumurgier J Bouillon K Rayana TB Schnitzler A. Social inequalities in multimorbidity, frailty, disability, and transitions to mortality: a 24-year follow-up of the Whitehall II cohort study. Lancet Public Health. (2020) 5:e42–50. doi: 10.1016/S2468-2667(19)30226-9 PMC7098476 31837974

[7] Jang AR Won CW Sagong H Bae E Park H Yoon JY. Social factors predicting improvement of frailty in community-dwelling older adults: Korean Frailty and Aging Cohort Study. Geriatr Gerontol Int. (2021) 21:465–71. doi: 10.1111/ggi.14160 33864343

[8] Kok RM Reynolds CF. Management of depression in older adults: A review. JAMA - J Am Med Assoc. (2017) 317:2114–22. doi: 10.1001/jama.2017.5706 28535241

[9] Rice F Riglin L Lomax T Souter E Potter R Smith DJ. Adolescent and adult differences in major depression symptom profiles. J Affect Disord. (2019) 243:175–81. doi: 10.1016/j.jad.2018.09.015 30243197

[10] Kroemer NB Opel N Teckentrup V Li M Grotegerd D Meinert S. Functional connectivity of the nucleus accumbens and changes in appetite in patients with depression. JAMA Psychiatry. (2022) 79:993–1003. doi: 10.1001/jamapsychiatry.2022.2464 36001327 PMC9403857

[11] Li L Wu C Gan Y Qu X Lu Z. Insomnia and the risk of depression: A meta-analysis of prospective cohort studies. BMC Psychiatry. (2016) 16(1):375. doi: 10.1186/s12888-016-1075-3 27816065 PMC5097837

[12] Vittengl JR Clark LA Thase ME Jarrett RB. Levels of depressed mood and low interest for two years after response to cognitive therapy for recurrent depression. Behav Res Ther. (2022) 148:103996. doi: 10.1016/j.brat.2021.103996 34775120 PMC8712398

[13] Sbolli M Fiuzat M Cani D O’Connor CM. Depression and heart failure: the lonely comorbidity. Eur J Heart Fail. (2020) 22:2007–17. doi: 10.1002/ejhf.1865 32468714

[14] Windle M Windle RC. Recurrent depression, cardiovascular disease, and diabetes among middle-aged and older adult women. J Affect Disord. (2013) 150:895–902. doi: 10.1016/j.jad.2013.05.008 23721922 PMC3759584

[15] Meza E Eng CW Sáenz JL Gilsanz P Glymour MM Torres JM. Elevated depressive symptoms and the risk of stroke among the Mexican older population. J Am Geriatr Soc. (2020) 68:2579–86. doi: 10.1111/jgs.16718 PMC7745730 32880905

[16] Andrade DMB Rocha RM Ribeiro ÍJS. Depressive symptoms among older adults with diabetes mellitus: a cross-sectional study. Sao Paulo Med J. (2023) 141(4):e2021771. doi: 10.1590/1516-3180.2021.0771.R5.09082022 PMC10065091 36197348

[17] Harshfield EL Pennells L Schwartz JE Willeit P Kaptoge S Bell S. Association between depressive symptoms and incident cardiovascular diseases. JAMA - J Am Med Assoc. (2020) 324(23):2396–405. doi: 10.1001/jama.2020.23068 PMC7739139 33320224

[18] Mitchell AJ Subramaniam H. Prognosis of depression in old age compared to middle age: a systematic review of comparative studies. Am J Psychiatry. (2005) 162(9):1588–601. doi: 10.1176/appi.ajp.162.9.1588 16135616

[19] Buigues C Padilla-Sánchez C Fernández Garrido J Navarro-Martínez R Ruiz-Ros V Cauli O. The relationship between depression and frailty syndrome: A systematic review. Aging Ment Health. (2015) 19:762–72. doi: 10.1080/13607863.2014.967174 25319638

[20] Robledo LMG Zepeda MUP. Complications of frailty. In: Frailty. Ruiz JG Theou O, editors. Cham: Springer (2024). doi: 10.1007/978-3-031-57361-3_8

[21] Vaughan L Corbin AL Goveas JS. Depression and frailty in later life: A systematic review. Clin Interv Aging. (2015) 10:1947–58. doi: 10.2147/CIA.S69632 PMC4687619 26719681

[22] Wang J Xu J Nie Y Pan P Zhang X Li Y. Effects of social participation and its diversity, frequency, and type on depression in middle-aged and older persons: evidence from China. Front Psychiatry. (2022) 13:825460. doi: 10.3389/fpsyt.2022.825460 35546944 PMC9085245

[23] Noguchi T Hayashi T Kubo Y Tomiyama N Ochi A Hayashi H. Association between Decreased Social Participation and Depressive Symptom Onset among Community-Dwelling Older Adults: A Longitudinal Study during the COVID-19 Pandemic. J Nutrition Health Aging. (2021) 25:1070–5. doi: 10.1007/s12603-021-1674-7 PMC8440728 34725663

[24] Glass TA Mendes De Leon C Marottoli RA Berkman LF. Population Based Study of Social and Productive Activities as Predictors of Survival among Elderly Americans. Available online at: www.bmj.com. 10.1136/bmj.319.7208.478 PMC28199 10454399

[25] Zunzunegui MV Alvarado BE Ser TD Otero A. Social networks, social integration, and social engagement determine cognitive decline in community-dwelling Spanish older adults. (2003). doi: 10.1093/geronb/58.2.S93 PMC3833829 12646598

[26] Tomaka J Thompson S Palacios R. The relation of social isolation, loneliness, and social support to disease outcomes among the elderly. J Aging Health. (2006) 18:359–84. doi: 10.1177/0898264305280993 16648391

[27] Lyu C Siu K Xu I Osman I Zhong J. Social isolation changes and long-term outcomes among older adults. JAMA Netw Open. (2024) 7:e2424519. doi: 10.1001/jamanetworkopen.2024.24519 39046736 PMC11270134

[28] Perlis RH Green J Simonson M Ognyanova K Santillana M Lin J. Association between social media use and self-reported symptoms of depression in US adults. JAMA Netw Open. (2021) 4:e2136213. doi: 10.1001/jamanetworkopen.2021.36113 PMC8611479 34812844

[29] Lin YC Yan HT. Association between political group participation and depressive symptoms among older adults: an 11-year longitudinal study in Taiwan. J Public Health (United Kingdom). (2022) 44:778–86. doi: 10.1093/pubmed/fdab335 PMC9715300 34498092

[30] Woo J Goggins W Sham A Ho SC. Social determinants of frailty. Gerontology. (2005) 51:402–8. doi: 10.1159/000088705 16299422

[31] Wang Y Chen Z Zhou C. Social engagement and physical frailty in later life: does marital status matter? BMC Geriatr. (2021) 21(1). doi: 10.1186/s12877-021-02194-x PMC8047563 33858354

[32] Zhao Y Hu Y Smith JP Strauss J Yang G. Cohort profile: The China health and retirement longitudinal study (CHARLS). Int J Epidemiol. (2014) 43:61–8. doi: 10.1093/ije/dys203 PMC3937970 23243115

[33] Zhao J Strauss G Yang J Giles P Hu Y Hu X. China health and retirement longitudinal study-2011-2012 national baseline users’ guide. (2013).

[34] Kojima G Iliffe S Walters K. Frailty index as a predictor of mortality: A systematic review and meta-analysis. Age Ageing. (2018) 47:193–200. doi: 10.1093/ageing/afx162 29040347

[35] Hao Q Sun X Yang M Dong B Dong B Wei Y. Prediction of mortality in Chinese very old people through the frailty index based on routine laboratory data. Sci Rep. (2019) 9(1). doi: 10.1038/s41598-018-36569-9 PMC6338748 30659252

[36] Searle SD Mitnitski A Gahbauer EA Gill TM Rockwood K. A standard procedure for creating a frailty index. BMC Geriatr. (2008) 8:24. doi: 10.1186/1471-2318-8-24 18826625 PMC2573877

[37] Romero-Ortuno R Kenny RA. The frailty index in Europeans: Association with age and mortality. Age Ageing. (2012) 41:684–9. doi: 10.1093/ageing/afs051 PMC3424051 22522775

[38] Fan J Yu C Guo Y Bian Z Sun Z Yang L. Frailty index and all-cause and cause-specific mortality in Chinese adults: a prospective cohort study. Lancet Public Health. (2020) 5:e650–60. doi: 10.1016/S2468-2667(20)30113-4 PMC7708389 33271078

[39] Lei X Sun X Strauss J Zhang P Zhao Y. Depressive symptoms and SES among the mid-aged and elderly in China: Evidence from the China Health and Retirement Longitudinal Study national baseline. Soc Sci Med. (2014) 120:224–32. doi: 10.1016/j.socscimed.2014.09.028 PMC4337774 25261616

[40] Fu H Si L Guo R. What is the optimal cut-off point of the 10-item center for epidemiologic studies depression scale for screening depression among Chinese individuals aged 45 and over? An exploration using latent profile analysis. Front Psychiatry. (2022) 13:820777. doi: 10.3389/fpsyt.2022.820777 35360127 PMC8963942

[41] Qin T Yan M Fu Z Song Y Lu W Fu A. Association between anemia and cognitive decline among Chinese middle-aged and elderly: evidence from the China health and retirement longitudinal study. BMC Geriatr. (2019) 19(1). doi: 10.1186/s12877-019-1308-7 PMC6849217 31718564

[42] Liu C Zhou R Peng X Zhu T Wei W Hao X. Relationship between depressive symptoms and anemia among the middle-aged and elderly: a cohort study over 4-year period. BMC Psychiatry. (2023) 23(1):572. doi: 10.1186/s12888-023-05047-6 37553590 PMC10408197

[43] Zhao Y Ye S Chen X Xia Y Zheng X. Polynomial Response Surface based on basis function selection by multitask optimization and ensemble modeling. Complex Intelligent Systems. (2022) 8:1015–34. doi: 10.1007/s40747-021-00568-7

[44] Rindskopf W. The value of latent class analysis in medical diagnosis. (1987) 6:. doi: 10.1002/sim.4780060412 3961312

[45] Dean N Raftery AE. Latent class analysis variable selection. Ann Inst Stat Math. (2010) 62:11–35. doi: 10.1007/s10463-009-0258-9 20827439 PMC2934856

[46] Asparouhov T Muthén B. Auxiliary variables in mixture modeling: three-step approaches using Mplus. Struct Equation Modeling. (2014) 21:329–41. doi: 10.1080/10705511.2014.915181

[47] Kim K Yoon H. Types of social engagement among older cancer survivors and the effect on depressive symptoms and life satisfaction: A latent class analysis. Oncol Nurs Forum. (2024) 51:25–37. doi: 10.1188/24.ONF.25-37 38108444

[48] Liu X Wang C Qiao X Phd S Phd YJ. Depression and Frailty among Chinese Community-Dwelling Older Adults. (2021). Available online at: https://doi.org/./j.gerinurse.

[49] Yaghi M Chatterjee S Alissawi F Daher M Khader Y Shawish MA. A cross-sectional survey of the prevalence and determinants of comorbid psychological distress in attendees at two general hospitals in Gaza. Lancet. (2021) 398:S52. doi: 10.1016/S0140-6736(21)01538-5 34227987

[50] Giannelis A Palmos A Hagenaars SP Breen G Lewis CM Mutz J. Examining the association between family status and depression in the UK Biobank. J Affect Disord. (2021) 279:585–98. doi: 10.1016/j.jad.2020.10.017 PMC7780845 33189065

[51] Brown SL Bulanda JR Lee GR. The significance of nonmarital cohabitation: marital status and mental health benefits among middle-aged and older adults. J Gerontol B Psychol Sci Soc Sci. (2005) 60(1):S21–9. doi: 10.1093/geronb/60.1.s21 15643043

[52] Wang H Kim K Burr JA Fingerman KL. Financial problems in established adulthood: implications for depressive symptoms and relationship quality with parents. J Adult Dev. (2023) 30:167–77. doi: 10.1007/s10804-022-09409-4 PMC9187927 35729889

[53] Evans MC Bazargan M Cobb S Assari S. Mental and physical health correlates of financial difficulties among african-american older adults in low-income areas of Los Angeles. Front Public Health. (2020) 8:21. doi: 10.3389/fpubh.2020.00021 32117856 PMC7028705

[54] Handley TE Rich J Lewin TJ Kelly BJ. The predictors of depression in a longitudinal cohort of community dwelling rural adults in Australia. Soc Psychiatry Psychiatr Epidemiol. (2019) 54:171–80. doi: 10.1007/s00127-018-1591-1 30155557

[55] Firth J Solmi M Wootton RE Vancampfort D Schuch FB Hoare E. A meta-review of “Lifestyle psychiatry”: the role of exercise, smoking, diet and sleep in the prevention and treatment of mental disorders. 10.1002/wps.20773 PMC7491615 32931092

[56] Dong L Xie Y Zou X. Association between sleep duration and depression in US adults: A cross-sectional study. J Affect Disord. (2022) 296:183–8. doi: 10.1016/j.jad.2021.09.075 34607059

[57] Brown PJ Roose SP O’Boyle KR Naqvi M Brickman AM Rutherford BR. Frailty and its correlates in adults with late life depression. Am J Geriatric Psychiatry. (2020) 28:145–54. doi: 10.1016/j.jagp.2019.10.005 PMC6997042 31734083

[58] Zhu J Zhou D Nie Y Wang J Yang Y Chen D. Assessment of the bidirectional causal association between frailty and depression: A Mendelian randomization study. J Cachexia Sarcopenia Muscle. (2023) 14:2327–34. doi: 10.1002/jcsm.13319 PMC10570069 37670569

[59] Lestari SK Eriksson M de Luna X Malmberg G Ng N. Frailty and types of social relationships among older adults in 17 European countries: A latent class analysis. Arch Gerontol Geriatr. (2022) 101. doi: 10.1016/j.archger.2022.104705 35461166

[60] Wang R Chen Z Zhou Y Zhang Z Wu X. Melancholy or Mahjong? Diversity, frequency, type, and rural-urban divide of social participation and depression in middle-and old-aged Chinese: A fixed-effects analysis. Soc Sci Med. (2019) 238:112518. doi: 10.1016/j.socscimed.2019.112518 31473574

[61] Menard C Pfau ML Hodes GE Kana V Wang VX Bouchard S. Social stress induces neurovascular pathology promoting depression. Nat Neurosci. (2017) 20:1752–60. doi: 10.1038/s41593-017-0010-3 PMC5726568 29184215

[62] Dudek KA Dion-Albert L Lebel M LeClair K Labrecque S Tuck E. Molecular adaptations of the blood-brain barrier promote stress resilience vs. depression. (2020) 117(6):3326–36. doi: 10.1073/pnas.1914655117/-/DCSupplemental PMC7022213 31974313

[63] Hashimoto Y Greene C Munnich A Campbell M. The CLDN5 gene at the blood-brain barrier in health and disease. Fluids Barriers CNS. (2023) 20(1). doi: 10.1186/s12987-023-00424-5 PMC10044825 36978081

