# Supplementary material for: The longitudinal study of the relationship between social participation pattern and depression symptoms in frail older adults
Source: Front Psychiatry. 2024 Sep 3;15:1440641. doi: 10.3389/fpsyt.2024.1440641 (PMC11405299; doi:10.3389/fpsyt.2024.1440641)
Supplement: Supplementary file 1 [file DataSheet1.docx]

Supplementary Material

# Supplementary Tables

| **Supplemental Table 1 Specific 40 items of frailty index construction** | | |
| --- | --- | --- |
| **No.** | **Items** | **Values** |
| 1 | Self-reported health | Very poor=1, Poor=0.8, Fair=0.6, Good=0.4, Very good=0.2 |
| 2 | Hypertension | Yes=1, No=0 |
| 3 | Dyslipidaemia | Yes=1, No=0 |
| 4 | Diabetes | Yes=1, No=0 |
| 5 | Chronic lung diseases | Yes=1, No=0 |
| 6 | Liver diseases | Yes=1, No=0 |
| 7 | Heart attack | Yes=1, No=0 |
| 8 | Stroke | Yes=1, No=0 |
| 9 | Kidney | Yes=1, No=0 |
| 10 | Stomach | Yes=1, No=0 |
| 11 | Emotional, nervous, or psychiatric problems | Yes=1, No=0 |
| 12 | Memory-related diseases | Yes=1, No=0 |
| 13 | Arthritis | Yes=1, No=0 |
| 14 | Asthma | Yes=1, No=0 |
| 15 | Physical disabilities | Yes=1, No=0 |
| 16 | Brain damage/intellectual disability | Yes=1, No=0 |
| 17 | Visual problem | Yes=1, No=0 |
| 18 | Hearing problem | Yes=1, No=0 |
| 19 | chronic pain | Yes=1, No=0 |
| 20 | Cancer | Yes=1, No=0 |
| 21 | Difficulty with getting up from a chair after sitting for a long period | Yes=1, No=0 |
| 22 | Difficulty with climbing stairs | Yes=1, No=0 |
| 23 | Difficulty with stooping/kneeling/crouching | Yes=1, No=0 |
| 24 | Difficulty with extending arms | Yes=1, No=0 |
| 25 | Difficulty with lifting weights over 5kg | Yes=1, No=0 |
| 26 | Difficulty with picking up a small coin from a table | Yes=1, No=0 |
| 27 | Difficulty with dressing | Yes=1, No=0 |
| 28 | Difficulty with bathing | Yes=1, No=0 |
| 29 | Difficulty with eating | Yes=1, No=0 |
| 30 | Difficulty with getting into or out of bed | Yes=1, No=0 |
| 31 | Difficulty with using toilet | Yes=1, No=0 |
| 32 | Difficulty with controlling urination and defecation | Yes=1, No=0 |
| 33 | Difficulty with doing household chores | Yes=1, No=0 |
| 34 | Difficulty with preparing hot meals | Yes=1, No=0 |
| 35 | Difficulty with shopping | Yes=1, No=0 |
| 36 | Difficulty with take medications | Yes=1, No=0 |
| 37 | Difficulty with handle finances | Yes=1, No=0 |
| 38 | Difficulty with telephoning | Yes=1, No=0 |
| 39 | Depression (measured CES-D-10^*^) | scores equal to or exceeding 10=1, other=0 |
| 40 | Cognitive impairment (measured by the TICS^*^) |  |
| *^*^Abbreviation: CES-D-10, 10-item short form of the Center for Epidemiologic Studies Depression Scale; TICS, Telephone Interview for Cognitive Status.* | | |

| **Supplemental Table 2 Specific problems of CES-D-10** | |
| --- | --- |
| Items | Scores |
| Total CES-D-10 scores | 0-30 |
| Somatic symptoms | 0-15 |
| I was bothered by things. | 0-3 |
| I had trouble keeping mind. | 0-3 |
| I felt everything I did was an effort. | 0-3 |
| My sleep was restless. | 0-3 |
| I Could not get going. | 0-3 |
| Depressed mood | 0-9 |
| I Felt depressed. | 0-3 |
| I Felt fearful. | 0-3 |
| I Felt lonely. | 0-3 |
| Positive emotion | 0-6 |
| I feel hopeful about the future. | 0-3 |
| I was happy. | 0-3 |
| *^*^Abbreviation: CES-D-10, 10-item short form of the Center for Epidemiologic Studies Depression Scale.* | |

| **Supplemental Table 3 Fit indices of the multi-LCA models for social participation patterns in 2015 and 2018** | | | | | | | | | | | | | | | | | |
| --- | --- | --- | --- | --- | --- | --- | --- | --- | --- | --- | --- | --- | --- | --- | --- | --- | --- |
| Model | | | AIC | | BIC | | | aBIC | | Entropy | | LMR(p) | | | BLRT(p) | | |
| 2015 | | |  | |  | | |  | |  | |  | | |  | | |
| 2-class | 18469.890 | | 18616.761 | | | 18543.677 | | | 0.996 | | | 0.000 | | | 0.000 | | |
| 3-class | 18132.336 | | 18355.836 | | | 18244.620 | | | 0.864 | | | 0.000 | | | 0.000 | | |
| 4-class | 17818.734 | | 18118.862 | | | 17969.515 | | | 0.899 | | | 0.000 | | | 0.000 | | |
| 5-class | 17682.790 | | 18059.547 | | | 17872.069 | | | 0.950 | | | 0.000 | | | 0.001 | | |
| 6-class | 17595.565 | | 18048.951 | | | 17823.341 | | | 0.963 | | | 0.000 | | | 0.002 | | |
| 7-class | 17529.541 | | 18059.556 | | | 17795.815 | | | 0.948 | | | 0.000 | | | 0.369 | | |
| 2018 | | |  | |  | | |  | |  | |  | | |  | | |
| 2-class | 14960.165 | | 15101.745 | | | 15028.663 | | | 0.997 | | | 0.000 | | | 0.000 | | |
| 3-class | 14694.396 | | 14909.844 | | | 14798.632 | | | 0.863 | | | 0.000 | | | 0.000 | | |
| 4-class | 14439.752 | | 14729.067 | | | 14579.726 | | | 0.896 | | | 0.000 | | | 0.000 | | |
| 5-class | 14332.352 | | 14695.536 | | | 14508.064 | | | 0.904 | | | 0.000 | | | 0.001 | | |
| 6-class | 14322.889 | | 14759.940 | | | 14534.339 | | | 0.979 | | | 0.000 | | | 0.379 | | |
| 7-class | 14210.899 | | 14721.818 | | | 14458.087 | | | 0.981 | | | 0.000 | | | 0.003 | | |
| **Supplemental Table 4 The specific distribution among different latent social participation classes in 2015** | | | | | | | | | | | | | | | | |  |
| Items | | | | | Class 1 Socially isolated 52.78% | Class 2 Socialize with friends 24.34% | | | Class 3 Board game enthusiast 6.14% | | Class 4 Helpful individual 11.31% | | | Class 5 Extensive social interaction 5.43% | | |  |
| Interacted with friends | | | | | 0.001 | 1.000 | | | 0.000 | | 0.627 | | | 0.306 | | |  |
| Played Ma-jong, played chess, played cards, or went to community club | | | | | 0.001 | 0.202 | | | 1.000 | | 0.225 | | | 0.297 | | |  |
| Provided help to family, friends, or neighbors who do not live with you | | | | | 0.000 | 0.000 | | | 0.000 | | 0.978 | | | 0.035 | | |  |
| Went to a sport, social, or other kind of club | | | | | 0.000 | 0.034 | | | 0.047 | | 0.101 | | | 0.589 | | |  |
| Took part in a community-related organization | | | | | 0.000 | 0.012 | | | 0.001 | | 0.060 | | | 0.122 | | |  |
| Done voluntary or charity work | | | | | 0.000 | 0.004 | | | 0.000 | | 0.048 | | | 0.039 | | |  |
| Cared for a sick or disabled adult who does not live with you | | | | | 0.000 | 0.010 | | | 0.004 | | 0.214 | | | 0.109 | | |  |
| Attended an educational or training course | | | | | 0.001 | 0.000 | | | 0.007 | | 0.010 | | | 0.030 | | |  |
| Stock investment | | | | | 0.001 | 0.001 | | | 0.000 | | 0.006 | | | 0.067 | | |  |
| Used the Internet | | | | | 0.001 | 0.035 | | | 0.006 | | 0.047 | | | 0.239 | | |  |
| None of these | | | | | 1.000 | 0.003 | | | 0.000 | | 0.003 | | | 0.005 | | |  |
|  | | | | |  |  | | |  | |  | | |  | | |  |
|  | | | | |  |  | | |  | |  | | |  | | |  |
| **Supplemental Table 5 The specific distribution among different latent social participation classes in 2018** | | | | | | | | | | | | | | | | |  |
| Items | | | | | Class 1 Socially isolated 51.59% | Class 2 Socialize with friends 24.46% | | | Class 3 Board game enthusiast 7.35% | | Class 4 Helpful individual 8.24% | | | Class 5 Extensive social interaction 8.36% | | |  |
| Interacted with friends | | | | | 0.001 | 1.000 | | | 0.000 | | 0.449 | | | 0.498 | | |  |
| Played Ma-jong, played chess, played cards, or went to community club | | | | | 0.001 | 0.217 | | | 1.000 | | 0.110 | | | 0.317 | | |  |
| Provided help to family, friends, or neighbors who do not live with you | | | | | 0.000 | 0.106 | | | 0.000 | | 1.000 | | | 0.298 | | |  |
| Went to a sport, social, or other kind of club | | | | | 0.000 | 0.018 | | | 0.073 | | 0.000 | | | 0.475 | | |  |
| Took part in a community-related organization | | | | | 0.000 | 0.007 | | | 0.000 | | 0.135 | | | 0.152 | | |  |
| Done voluntary or charity work | | | | | 0.000 | 0.001 | | | 0.000 | | 0.018 | | | 0.073 | | |  |
| Cared for a sick or disabled adult who does not live with you | | | | | 0.000 | 0.008 | | | 0.006 | | 0.135 | | | 0.124 | | |  |
| Attended an educational or training course | | | | | 0.001 | 0.000 | | | 0.004 | | 0.000 | | | 0.011 | | |  |
| Stock investment | | | | | 0.001 | 0.001 | | | 0.000 | | 0.000 | | | 0.038 | | |  |
| Used the Internet | | | | | 0.001 | 0.033 | | | 0.011 | | 0.006 | | | 0.159 | | |  |
| None of these | | | | | 1.000 | 0.005 | | | 0.000 | | 0.003 | | | 0.000 | | |  |

| **Supplement Table 6 The outcomes produced by RMM after incorporating predictive variables.** | | | | |
| --- | --- | --- | --- | --- |
|  | Estimate | S.E. | Est./S.E. | P-Value |
| Age |  |  |  |  |
| Class 2 | 0.005 | 0.006 | 0.812 | 0.417 |
| Class 3 | -0.015 | 0.010 | -1.413 | 0.000 |
| Class 4 | -0.039 | 0.008 | -4.869 | 0.000 |
| Class 5 | -0.055 | 0.014 | -4.000 | 0.000 |
|  |  |  |  |  |
| Gender |  |  |  |  |
| Class 2 | 0.212 | 0.08 | 2.655 | 0.008 |
| Class 3 | -0.648 | 0.137 | -4.744 | 0.000 |
| Class 4 | -0.205 | 0.103 | -1.990 | 0.047 |
| Class 5 | 0.416 | 0.167 | 2.487 | 0.013 |
| FI |  |  |  |  |
| Class 2 | -1.095 | 0.303 | -3.615 | 0.000 |
| Class 3 | -3.923 | 0.596 | -6.577 | 0.000 |
| Class 4 | -2.293 | 0.435 | -5.265 | 0.000 |
| Class 5 | -3.784 | 0.708 | -5.345 | 0.000 |
| *^*^Abbreviation: RMM, regression mixture model; FI, frailty index; S.E., standard error.* | | | | |

| **Supplement Table 7 The results of RMM after including depression severity as the outcome variable** | | | | | | | |
| --- | --- | --- | --- | --- | --- | --- | --- |
|  |  | Prob | S.E. | OddsRatio | S.E. | 2.50%C.I. | 97.50%C.I. |
| Class 1 |  |  |  |  |  |  |  |
|  | NDS group | 0.395 | 0.010 | 1.000 | 0.000 | 1.000 | 1.000 |
|  | DS group | 0.501 | 0.010 | 1.000 | 0.000 | 1.000 | 1.000 |
|  | DD group | 0.104 | 0.006 | 1.000 | 0.000 | 1.000 | 1.000 |
| Class 2 |  |  |  |  |  |  |  |
|  | NDS group | 0.435 | 0.017 | 1.000 | 0.000 | 1.000 | 1.000 |
|  | DS group | 0.477 | 0.017 | 0.866 | 0.073 | 0.735 | 1.020 |
|  | DD group | 0.087 | 0.009 | 0.765 | 0.109 | 0.579 | 1.011 |
| Class 3 |  |  |  |  |  |  |  |
|  | NDS group | 0.520 | 0.037 | 1.000 | 0.000 | 1.000 | 1.000 |
|  | DS group | 0.436 | 0.037 | 0.662 | 0.106 | 0.484 | 0.906 |
|  | DD group | 0.044 | 0.015 | 0.326 | 0.119 | 0.160 | 0.666 |
| Class 4 |  |  |  |  |  |  |  |
|  | NDS group | 0.425 | 0.028 | 1.000 | 0.000 | 1.000 | 1.000 |
|  | DS group | 0.493 | 0.026 | 0.916 | 0.113 | 0.719 | 1.167 |
|  | DD group | 0.083 | 0.013 | 0.741 | 0.152 | 0.496 | 1.108 |
| Class 5 |  |  |  |  |  |  |  |
|  | NDS group | 0.527 | 0.032 | 1.000 | 0.000 | 1.000 | 1.000 |
|  | DS group | 0.418 | 0.031 | 0.627 | 0.087 | 0.477 | 0.823 |
|  | DD group | 0.054 | 0.014 | 0.391 | 0.114 | 0.221 | 0.693 |
| *^*^Abbreviation: RMM, regression mixture model; S.E., standard error; NDS, non-depressive symptom; DS, depressive symptom; DD, depressive disorder.* | | | | | | | |

#
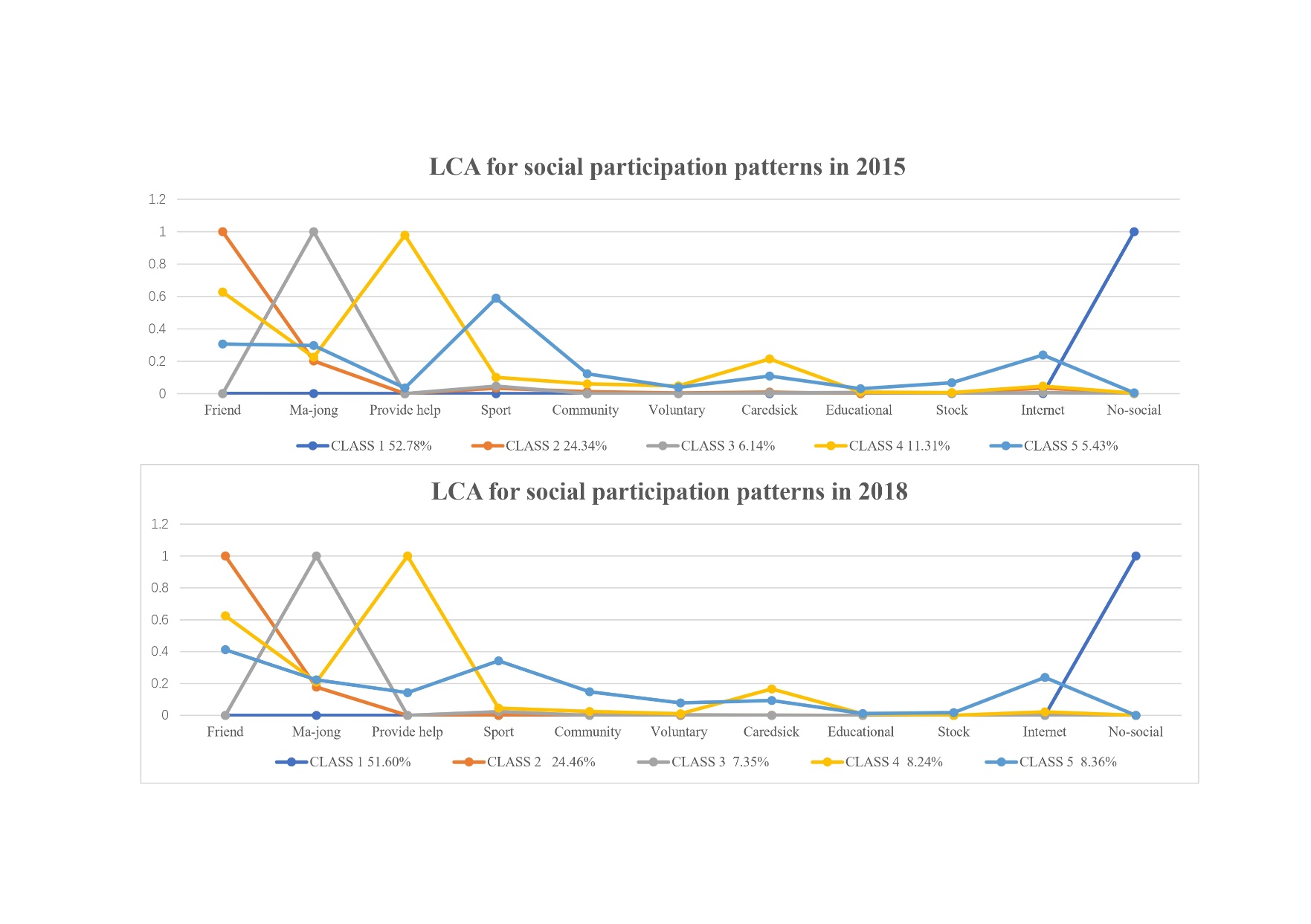
Supplementary Figures

**Supplementary Figure 1 The result of LCA for social participation patterns in 2015 and 2018**
